# Academic Orthopaedics As a Driver of Gender Diversity in the Orthopaedic Workforce: A Review of 4,519 Orthopaedic Faculty Members

**DOI:** 10.5435/JAAOSGlobal-D-21-00028

**Published:** 2022-02-03

**Authors:** Benjamin Kuhns, Brittany E. Haws, Shannon Kaupp, Michael D. Maloney, Emily E. Carmody, Sandeep Mannava

**Affiliations:** From the Department of Orthopaedics & Rehabilitation, University of Rochester Medical Center, Rochester, NY (Dr. Kuhns, Dr. Haws, Dr. Maloney, Dr. Carmody, and Dr. Mannava), and Upstate Medical University, Syracuse, NY (Dr. Kaupp).

## Abstract

The purpose of this study was to perform a cross-sectional analysis on the gender composition of practicing academic orthopaedic surgeons using three databases composed of clinical orthopaedic surgeons.

**Methods:**

A comprehensive database of 4,519 clinically active academic orthopaedic surgeons was compiled for this study after the review of publicly available data. The complied data set was evaluated alongside orthopaedic databases obtained from the 2017 Association of American Medical Colleges (AAMC) Faculty Administrative Management Online User System and the 2016 American Academy of Orthopaedic Surgery (AAOS) Orthopaedic Practice in the US census representing the entire academy membership orthopaedic workforce. Gender status was obtained and compared between the three databases.

**Results:**

Of the 4,519 clinically active academic orthopaedic surgeons analyzed, 435 (10%) were female compared with 19% for the AAMC faculty database and 7% for the AAOS members. Fourteen percent of women had achieved the rank of professor compared with 25% of the men (*P* < 0.001). AAMC faculty had a significantly higher percentages of female representation compared with both the clinical faculty (19% versus 10%; *P* < 0.001) and AAOS members (19% versus 7%; *P* < 0.001).

**Conclusion:**

Despite multiple initiatives designed to increase diversity, the 7% female representation in the orthopaedic workforce identified in this study remains markedly lower than other medical and surgical specialties. A higher percentage of women were associated with the AAMC orthopaedic faculty compared with clinically active female surgeons at these institutions. Academic orthopaedic surgeons had greater female representation than the general orthopaedic workforce, highlighting the importance of training institutions at promoting gender equity.

Gender diversity in orthopaedic surgery lags behind most other medical specialties.^1-3^ Despite increases in female representation over the past 20 years, only 20% of the academic orthopaedic faculty are women, which is the lowest of all recorded medical and surgical subspecialties.^4^ In addition, academic faculty data incorporate all orthopaedic faculty members, including nonoperative and research faculty, making the number of clinically active female academic orthopaedic surgeons considerably less. Furthermore, when viewing the field of orthopaedics as a whole, the diversity gap is increasingly stark with only 7.6% of the female clinically active American Academy of Orthopaedic Surgery (AAOS) members according to the most recent Orthopaedic Practice in the United States (OPUS) census.^5^ An orthopaedic work force that is representative of general US population demographics is beneficial to providing optimal and culturally competent medical care for the increasingly diverse US patient population.^5,6^ To address these issues, the American Orthopaedic Association and AAOS, as well as subspecialty groups have organized multiple task forces and seminars designed to improve gender diversity within the orthopaedic profession.

Increasing diversity in the orthopaedic workforce directly benefits both patients and surgeons by increasing access to orthopaedic care,^7^ reducing disparities in the provision of care,^6,8^ and improving the training environment of orthopaedic residents and fellows.^9^ Although the underrepresentation of women in orthopaedic residency programs has been recently studied, the gender composition of academic and nonacademic faculty requires additional evaluation.^10,11^ The purpose of this study was to perform a cross-sectional analysis on the sex of clinically active academic orthopaedic surgeons compared with the academic orthopaedic faculty and broader orthopaedic population. Because academic institutions have been leaders in advancing the field, we hypothesize that academic orthopaedic surgery will have increased representation of female surgeons compared with the general orthopaedic workforce.

## Methods

### Data Acquisition

This report was a cross-sectional study of publicly available data from self-reported institutional websites, making it exempt from institutional review board approval. A clinically active academic orthopaedic surgeon database was created through the review of public orthopaedic departmental websites for all 175 Accreditation Council for Graduate Medical Education–accredited orthopaedic residency training programs. Inclusion criteria required subjects to be practicing orthopaedic surgeons listed as residency faculty on the department website at the time of data accrual (September 2018). Research faculty and nonorthopaedic physicians (ie, neurosurgery, nonoperative sports medicine, anesthesiology, physical medicine, and rehabilitation) listed under the department of orthopaedics were excluded. Population characteristics, including sex, academic rank, leadership position, years in practice, and program population, were recorded where available. Academic rank was defined by four categories: instructor, assistant professor, associate professor, and professor to be consistent with the Association of American Medical Colleges (AAMC) terminology. Leadership roles included department chair (including medical director and institution CEO), residency program director, or chief/director of a subspecialty department.

This database was then compared with aggregate demographic data collected through the AAMC Faculty Administrative Management Online User System database and the 2016 OPUS orthopaedic surgical census.^12,13^ The OPUS data set provides data for the orthopaedic workforce and is comprised survey responses from 7,704 practicing orthopaedic surgeons of 29,613 members surveyed (26%). The AAMC data set represents a deidentified aggregate cohort of academic medical school faculty organized by department (ie, general surgery, orthopaedic surgery, and internal medicine) and includes a combination of clinical and nonclinical faculty and researchers from 149 participating medical schools. For ease of comparison, the data sets are labeled “Clinical Faculty,” “AAMC Faculty,” and “AAOS Members” representing the individually compiled clinical faculty database, the 2017 AAMC academic orthopaedic faculty database, and the 2016 OPUS database, respectively.

### Statistical Analysis

Data were analyzed with JMP version 14.0. Demographic variables were assessed with either percentages or means and standard deviations where appropriate. Continuous variables were compared with a bivariate regression analysis. Categorical and continuous data were compared with a one-way analysis of variance. Categorical variables and proportional analysis were analyzed with Pearson χ^2^ correlations. Significance was determined by a *P* < 0.05 with highly significant results reported as *P* < 0.001.

## Results

### Clinical Faculty

Of the 4,519 orthopaedic surgeons included in the clinical faculty cohort, 4,084 were male (90%) and 435 were female (10%). Combined, there were 543 leaders comprising chairmen, medical directors, division chiefs, and program directors and 764 professors of orthopaedic surgery. Male surgeons were more likely to have leadership roles (13% versus 8%; *P* = 0.003) and professorships (25% versus 14% *P* < 0.0001) than female surgeons (Table 1). Of all the professors (n = 764), only 49 (6.4%) were female, whereas of the leaders (543), only 33 (6.1%) were female. Female surgeons had a significantly lower rate of holding chair appointments (0.2% [1/435] versus 3.7% [152/4,084]; *P* = 0.001), but there were no proportional gender differences between chiefs or program directors (7.3% versus 8.7%; *P* = 0.32). In addition, male surgeons had significantly more years in practice than female surgeons (19.1 ± 11.9 versus 11.8 ± 8.2), with a greater likelihood of having greater than 10 years in practice (Table 1).

**Table 1:** Gender Differences in Practice Duration and Rates of Holding Leadership Positions or Professor Status

Factor	Female (n = 435), n (%)	Male (n = 4,084), n (%)	*P* Value
Leadership position	33 (8)	510 (13)	0.0028
Chair	1 (0.2)	152 (3.7)	0.0001
Chief/program director	32 (7.3)	355 (8.7)	0.32
Professor	49 (14)	715 (25)	<0.0001
Practice >10 years	204 (50)	2,792 (73)	<0.0001

### Association of American Medical Colleges Faculty

**Figure 1:**
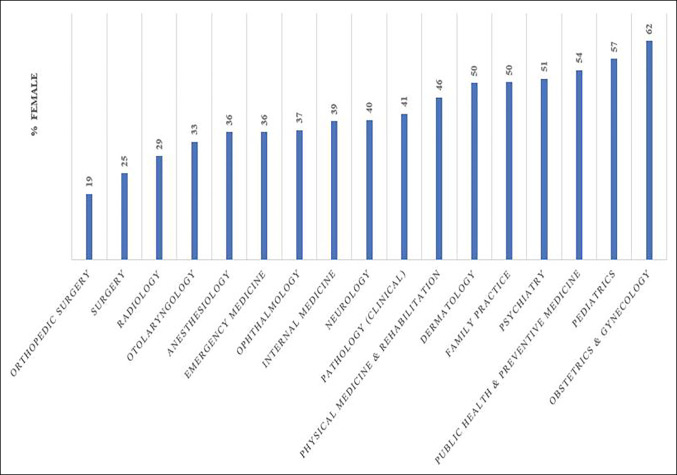
Graph showing female physician representation by department according to the AAMC faculty database.^13^ Orthopaedic surgery had a significantly (*P* < 0.0001) lower proportion of female physicians compared with all other specialties. AAMC = American Academy of Medical Colleges.

The AAMC faculty database reported orthopaedic surgery as having the lowest percentage of female academic faculty of any subspecialty (19%; Figure 1), including general surgery (24.5%; *P* < 0.0001), anesthesiology (36%; *P* < 0.0001), and internal medicine (39%; *P* < 0.0001). In total, 19% of the female representation (790/4,139) among AAMC orthopaedic faculty members was significantly higher than the 10% found among clinically active academic orthopaedic faculty (*P* < 0.001).

### American Academy of Orthopaedic Surgery Members

**Figure 2:**
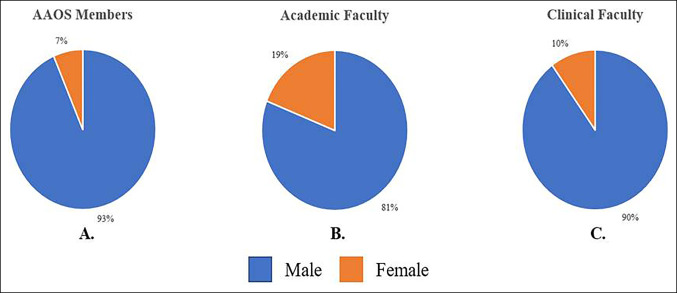
Pie chart showing gender distribution in orthopaedics across 2016 AAOS members (**A**), 2017 AAMC orthopaedic faculty (**B**), and 2018 clinical faculty (**C**). AAMC = American Academy of Medical Colleges, AAOS = American Academy of Orthopaedic Surgeons.

Of 7,704 AAOS member respondents, 93.4% were male compared with 6.5% female with 0.1% not recording a sex (Figure 2, A). This was significantly lower than the 2017 AAMC faculty cohort (Figure 2, B: 19% female; *P* < 0.0001) and the clinical faculty cohort (Figure 2, C: 10% female; *P* < 0.0001).

## Discussion

In 2018, we performed a cross-sectional study of 4,519 clinically active orthopaedic faculty in 175 different orthopaedic residency training programs evaluating differences in demographic representation. We compared this database with publicly available 2017 AAMC medical school faculty rosters and the 2016 OPUS census of AAOS members. We found that clinical orthopaedic faculty were predominantly male (90%), which was consistent with the AAMC and AAOS databases, as well as previously reported data regarding gender disparities in orthopaedic surgery.^1,2,14^ We also found that clinically active academic orthopaedic surgeons and AAMC academic faculty had increased representation of women compared with AAOS members.

Consistent with previous reports, we found orthopaedic surgery to have the lowest female representation compared with any other medical or surgical specialty.^14,15^ A recent study by Okike et al^11^ using the AAMC Faculty Administrative Management Online User System database from 2014 to 2016 identified a 17.6% (range 0% to 46.3%) rate of female composition among academic orthopaedic faculty. They also found that female medical students attending institutions with higher resident and faculty gender diversity in orthopaedics were more likely to apply for an orthopaedic residency.^11^ This is similar to the 19% female representation found in this study through the 2017 faculty roster and reflects the gradually increasing percentage of female academic orthopaedic faculty over the past decade.^14^ The discrepancy in female representation found between AAMC faculty (19%) and the clinical faculty database (10%) may result from increasing female representation in research or other nonsurgical roles. This is supported in a report by Daniels et al who found that a 16.7% female representation in orthopaedic research faculty compared with 8.1% of the clinical faculty in 2010.^3^ As such, the data available from the AAMC on female representation in orthopaedic surgery may be inflated compared with the number of clinically active female surgeons. Despite this, when comparing both the AAMC and the clinical faculty databases with the OPUS census data, we found markedly increased female representation in academic orthopaedics compared with the general workforce. The increased representation of female surgeons in academic orthopaedics highlights the importance of teaching institutions in promoting systemic change to improve gender representation throughout the field.

Previous literature has highlighted the lack of female representation particularly in leadership positions. Among all medical specialties, women have been reported to comprise only 15.8% of the department chairs with minimal representation (0% to 1%) among orthopaedic department chairs.^16^ Similarly, we found a markedly low rate of female surgeons achieving chair positions; however, we found no proportional differences between male and female representation among department chiefs or program directors. When evaluating academic professional advancement, we found that women were proportionally less likely to be professors compared with their male counterparts. Of all women faculty members in orthopaedic surgery, only 14% of the women (49/435) achieved the rank of professor, compared with 25% of the men (715/4,084) (*P* < 0.01). In addition, when examining the percentage of women achieving the rank of professor for all orthopaedic faculty (both women and men), only 6.7% of the orthopaedic professors (49/764) were female. This represents a roughly two-fold increase in representation compared with the 3.8% reported by Day et al^14^ in 2007 but lags behind the current 25% of the female professorship (9,874/38,478) reported by the most recent 2019 AAMC data. The decreased female representation among full professors may be associated with an overall shorter time in practice to date; however, future longitudinal studies must be done to evaluate female advancement in orthopaedics over time.

Female representation in leadership is important to appropriately support measures to increase diversity in the workforce. It also plays a critical role in the continued recruitment of women into the field. Previous studies have demonstrated that residency programs with the most female trainees have higher numbers of female faculty and greater percentages of women in leadership positions.^17^ In one report by Van Heest et al,^1^ programs with high percentages of female residents were found to have three to four women on faculty with 40% holding leadership roles. In addition, women are more likely than their male counterparts to indicate having same-sex role models positively influenced their choice to pursue orthopaedics.^18^ The availability of female mentors and leaders is instrumental in encouraging female students to pursue a career in orthopaedics and further improving gender equity. Multiple initiatives have been developed over the years to address the need for female mentorship and have been relatively successful in improving gender equity. Among these, the Ruth Jackson Orthopaedic Society was created as a networking organization for female orthopaedic surgeons and provides substantial support to its members through formal mentorship, research grants, awards, and traveling fellowships. The Ruth Jackson Orthopaedic Society has a proven track record of increasing female recruitment into orthopaedics with a recent study demonstrating that 80% of the medical student scholarship winners in the society went on to a career in orthopaedics compared with 45% of the medical student society members who were not selected for a scholarship.^19^

Focused outreach and mentorship opportunities during medical school is another method of increasing the ranks of female orthopaedic surgeons. The Perry Initiative is an organization devoted to increasing female representation through the Medical Student Outreach Program—a 3-hour exposure session for female medical students, including both lecture and hands on based instruction.^20^ An additional program sponsored by the Perry Initiative focuses on recruiting high school junior and senior women to orthopaedics and engineering. Nth Dimensions is another program designed to increase representation of women and underrepresented minorities in orthopaedics through mentorship, clinical and research opportunities, and professional development. The comprehensive summer internship associated with the Nth Dimensions program has shown that female participants were 43.2 times more likely to apply to orthopaedic residency positions compared with national controls (*P* < 0.001).^21^ In addition to providing increased exposure and mentorship to female medical students, these initiatives can help dispel perceptions of physical requirements and poor work/life balance, as well as combat gender discrimination at the medical school, residency, and faculty levels.^22,23^ Given that 49.6% of the enrolled medical students were female in 2018 to 2019, the gender gap in orthopaedics continues to persist despite modest improvement during the past 20 years.^18^ Over the coming decades, the role of academic orthopaedic surgery in recruiting female candidates to residency, retaining academic female surgeons, and promoting women in the general (nonacademic) orthopaedic workforce will remain paramount.

There are multiple limitations present in this study. This is an observational cross-sectional study of the orthopaedic workforce encompassing three databases collected at separate points in time (Clinical Faculty Database: 2018; AAMC Faculty Database: 2017; AAOS Members Database: 2016). Thus, we were unable to evaluate for longitudinal trends and could only compare the present data with previous reports. At the time of data analysis and manuscript preparation, the 2016 OPUS census was the most recent available. Although the 2018 census has recently been published, the increase in female representation from 6.5% to 7.6% did not alter the significance of our findings in this study. Although the AAOS census had data on 7,704 surgeons, this Clinical Faculty Database compiled and analyzed for this study only comprised 26% AAOS members and therefore may not accurately represent the entirety of the orthopaedic workforce. It is possible that female AAOS members had proportionally fewer responses than male members, thus artificially inflating the ratio of male to female surgeons. An additional limitation was the use of sex compared with gender identity, which was not considered in this study. Despite these limitations, we feel that the comprehensive nature of this study, including an analysis of an individualized database of 4,519 clinically practicing academic orthopaedic surgeons and available AAMC faculty, and AAOS data will provide valuable information on the current representation of women in the academic and general orthopaedic workforce.

## Conclusion

Consistent with previous studies, we found that the orthopaedic profession is predominantly male; however, female representation is markedly increased in the academic setting compared with the total workforce. Greater female representation was observed among the AAMC orthopaedic faculty compared with clinically active female surgeons. Academic orthopaedic surgery departments with residency training programs should continue to promote diversity enhancing initiatives to both recruit and retain underrepresented surgeons in efforts to combat inequities in the provision of care.
